# Why Aromaticity Is a Suspicious Concept? Why?

**DOI:** 10.3389/fchem.2017.00022

**Published:** 2017-03-24

**Authors:** Miquel Solà

**Affiliations:** Institut de Química Computacional i Catàlisi and Departament de Química, Universitat de GironaGirona, Spain

**Keywords:** aromaticity, conjugation, hyperconjugation, multidimensional character, indicators of aromaticity

From time to time I have the opportunity to give lectures on topics related to aromaticity. Quite often in these occasions I get comments from the audience complaining about the fact that aromaticity is not a well-defined concept. My usual answer is that the most fruitful concepts in chemistry share the same lack of strict definition (Grunenberg, 2017). In one of these occasions, the comment was formulated by someone who give a talk the day before justifying all the results he/she obtained using the concept of hyperconjugation. His/her comment was a little bit irritating to me because, in a way, he/she was saying I am a serious scientist because I am working with rigorous concepts like hyperconjugation whereas you are a kind of pseudoscientist playing with floppy concepts like aromaticity. Was he/she right? I do not think so.

Conjugation involves interactions (electron delocalization) between π-orbitals, although its definition can also be extended to p-orbitals to cover lone pair interactions with the π-system. Hyperconjugation accounts for the interaction between two orbitals with π-symmetry where one or both of them come from a saturated moiety (Mulliken, 1939; Mulliken et al., 1941). It can also be defined as the interaction between the orbitals involved in a σ-bond (usually C–H or C–C) with those related with an adjacent π-bond (usually C=C) or another σ-bond. Aromaticity is conjugation (and in some cases hyperconjugation) that generates closed two- and three-dimensional electronic circuits. Conjugation, hyperconjugation, and aromaticity lead to stabilizing interactions that influence the geometry, electron density, dissociation energies or nuclear magnetic resonance properties among many other physicochemical observables. Despite their importance and widespread use, neither hyperconjugation nor aromaticity have a strict physical definition and, therefore, these properties cannot be experimentally directly measured. These two properties share the same origin that is stabilization due to electron delocalization. Indeed, differences between these two concepts are minor as compared to similarities. Thus, the claim that one property is more rigorous than the other is totally unfounded.

The above-mentioned anecdote together with the existence of a series of papers (Balaban, 1980; Lloyd, 1996; Hoffmann, 2015) discussing the concept of aromaticity point out that aromaticity for some chemists is a controversial concept, while parent concepts like conjugation or hyperconjugation are not. Why? In the next paragraphs, I pointed out possible explanations to this fact and I propose ways of action to improve the prestige of this concept.

## Possible reasons for the low reputation of the aromaticity concept

The reasons for aromaticity being perceived by some members of the chemical community as questionable are:

### Too many aromaticity descriptors

Probably, the problem with aromaticity is not the concept itself, but the way aromaticity is characterized. No single property exists that could be taken as a direct measure of aromaticity. The evaluation of global or local aromaticity of a molecule is usually done indirectly by measuring some physicochemical property that reflects a manifestation of its aromatic character. This leads to a large number of classical structural (Krygowski and Cyrański, 2001; Krygowski et al., 2014), magnetic (Mitchell, 2001; Chen et al., 2005; Gershoni-Poranne and Stanger, 2015), energetic (Cyrański, 2005), electronic (Poater et al., 2005; Feixas et al., 2015), and reactivity-based (Mucsi et al., 2007) measures of aromaticity. In my opinion, there are too many indicators of aromaticity. An extensive but not exhaustive list (Solà, 2017) of acronyms includes ASE, RE, ISE, AI, HOMA, Julg index, Jug index, Bird index, PDI, FLU, FLU_π_, MCI, I_ring_, I_NG_, I_NB_, EDDB, AV1245, ELF_π_, ATI, θ, PLR, η, ρ_RCP_, ∧, ACID, ARCS, NICS, NICS_zz_, NICS_π_, NICS-XY-scan…The problem is that not all of them give the same ordering by aromaticity of a series of rings or molecules adding confusion to the field (Balaban, 1980; Lloyd, 1996; Poater et al., 2004). And the reason is that all indices are just approximations to the problem of measuring aromaticity and some of them are not useful for certain situations (for instance, to detect the aromaticity of transition states in cycloaddition reactions). Other indices are simply not good enough to provide reliable results. This is particularly the case if we want to quantify the aromaticity of metalloaromatic compounds (Feixas et al., 2013) because most of the methods to measure aromaticity were developed for the classical aromatic organic molecules and they cannot be directly applied to metallic clusters. For these species, the electronic indices and magnetic measures are likely the best choices (Feixas et al., 2010).

### The multidimensional character of aromaticity

In relation with the previous point, many times the apparent contradictions found among differently based indices are overcome by addressing to the so-called multidimensional character of aromaticity (Katritzky et al., 1989, 1998, 2001; Jug and Köster, 1991; Krygowski and Cyrański, 2001; Cyrański et al., 2002). According to this view, different indices afford divergent orderings since one compound may be more aromatic than other in one dimension and less aromatic in another (Krygowski et al., 2000). The problem with this view is that, in principle, one could get any ordering of aromaticity provided one looks at the correct direction in the multidimensional space. If we accept the multidimensional character of aromaticity, then this concept becomes quite useless because one can justify almost any result. Some years ago, Bultinck et al. (Bultinck et al., 2006; Bultinck, 2007) already warned about the use of the multidimensional character of aromaticity as a generic excuse to consider any aromaticity index a good descriptor of aromaticity irrespective of the results obtained.

### Too many types of aromaticity

The list of different types of aromaticity is also very broad (Solà, 2017). Among them we can cite Hückel aromaticity, Möbius aromaticity, excited state aromaticity, Hückel-Baird hybrid aromaticity, homoaromaticity, heteroaromaticity, claromaticity, three-dimensional aromaticity, spherical aromaticity, cubic aromaticity, octahedral aromaticity, σ-aromaticity, δ-aromaticity, multiple aromaticity, conflicting aromaticity, metalloaromaticity, chelatoaromaticity, quasi-aromaticity, transition state aromaticity, hyperaromaticity…The adjective added to aromaticity in each case helps to describe a particular situation. In a sense, they are totally justified, however, the existence of so many types of aromaticity can be perceived as hectic.

### The unwarranted use of aromaticity

The link between aromaticity and stability is well-established. However, among a series of isomers or different electronic states, not always the most stable is the most aromatic. Aromaticity is just one more of the many factors that affect the relative energies of isomers. Other aspects, such as strain energy, hyperconjugation, the presence of hydrogen bonds, long-range interactions, and so forth, may have, in many cases, a greater influence. Aromaticity cannot explain all observed differences. It should not come as a surprise to find isomerization energies that cannot be justified from the different aromatic character of the isomers. For instance, N-substituted imidazoles are more stable than the corresponding pyrazoles although the higher stability of imidazoles is not due to the higher aromaticity of imidazole rings but a weaker N–N bond found in the pyrazole rings (Curutchet et al., 2011).

## Possible solutions and recommendations

Possible recommendations that may help to improve the prestige of aromaticity are:

### Propose new descriptors of aromaticity only when justified

As said before, there are too many indicators of aromaticity. New indicators are welcome but only when they improve the currently existing ones because they are cheaper to compute and have a similar quality to those previously defined or they have a better performance. To assess the quality of the descriptors, the best option is to employ a set of aromaticity tests (Feixas et al., 2008; Solà et al., 2010) that are designed based on the accumulated chemical experience about the expected aromaticity trends in a given series of compounds. The larger the number of tests passed, the better the indicator (Feixas et al., 2008, 2010; Solà et al., 2010).

### Avoid using the excuse of the multidimensional character of aromaticity

Available indices of aromaticity do not always give consistent results among themselves (Balaban, 1980; Lloyd, 1996; Poater et al., 2004; Zhao et al., 2017). Thus, for instance, predictions based on magnetic criteria of aromaticity often deviates from those based on energetic grounds (Aihara, 2006). In some cases, contradictions found among indices are justified by addressing to the so-called multidimensional character of aromaticity (Katritzky et al., 1989, 1998, 2001; Jug and Köster, 1991; Krygowski and Cyrański, 2001; Cyrański et al., 2002). Recent works, however, have proved that, in many cases, the contradictions between indices are due to failures of some indicators to correctly measure aromaticity and that the multidimensional character of aromaticity is not fully founded (Bultinck et al., 2006; Bultinck, 2007; Badri and Foroutan-Nejad, 2016).

### Using a set of aromaticity indicators

In general, it is advisable to use a set of indices based on different physicochemical properties to characterize aromatic compounds (Hoffmann et al., 1997; Poater et al., 2004; Chen et al., 2005; Tsipis, 2005; Van Droogenbroek et al., 2005; Feixas et al., 2007). When using a large set of aromaticity indicators, conclusions reached are much more reliable. In particular, one can feel especially safe about the derived conclusions when all criteria provide the same results for the set of compounds analyzed. If different criteria produce contradictory results, the conclusions, if any, are necessarily weaker.

### Avoid defining new types of aromaticity

As said before, the number of different types of aromaticity is probably too large. In some cases, the use of adjectives in front of the word aromaticity can be helpful. In other occasions, it is unnecessary. For instance, when we write that pyridine is a heteroaromatic molecule or when we classify metallabenzenes as metalloaromatic, we are not giving any extra information. By just calling them aromatic, it would be enough. In fact, no one considers that the Zn–Zn bond (Zhu et al., 2006) is a metallocovalent bond. We simply say that it is a covalent bond. When naming aromatic compounds, one should keep nomenclature as simple as possible.

In summary, since the discovery of benzene in 1825, aromaticity has been one of the most vexing yet fascinating concepts in chemistry. It is useful to understand the structure, stability, spectroscopy, magnetic properties, and chemical reactivity of many compounds. Like many fruitful concepts in chemistry, aromaticity is vaguely defined. However, this lack of a strict definition is not a reason strong enough to question the concept. In this article, we have listed a series of good practices that may improve the perception of this concept.

## Author contributions

The author confirms being the sole contributor of this work and approved it for publication.

### Conflict of interest statement

The author declares that the research was conducted in the absence of any commercial or financial relationships that could be construed as a potential conflict of interest.
